# Ecological and Statistical Evaluation of Genetic Algorithm (GARP), Maximum Entropy Method, and Logistic Regression in Predicting Spatial Distribution of *Astragalus* sp.

**DOI:** 10.1155/sci5/4003408

**Published:** 2025-03-31

**Authors:** Amir Ghahremanian, Abbas Ahmadi, Hamid Toranjzar, Javad Varvani, Nourollah Abdi

**Affiliations:** ^1^Department of Natural Resources and Environment, Ar.C., Islamic Azad University, Arak, Iran; ^2^Food Security Research Centre, Ar.C., Islamic Azad University, Arak, Iran; ^3^Research Center of Applied Plant Science, Ar.C., Islamic Azad University, Arak, Iran

**Keywords:** *astragalus* sp., genetic algorithm, logistic regression, MaxEnt

## Abstract

This study aims to evaluate the potential habitat of *Astragalus* sp. using three different species distribution modeling methods: the maximum entropy (MaxEnt) model, the Genetic Algorithm for Rule-Set Production (GARP), and logistic regression. The primary objective was to identify key environmental factors that influence the spatial distribution of *Astragalus* sp. in the Savar-Abad basin's rangelands. Vegetation sampling was carried out across diverse vegetation types within the study area, using 2–10 square meter plots to capture a representative sample of plant species distribution. Soil sampling was conducted at varying depths to capture essential soil properties, including physical (clay, gravel, silt, and sand) and chemical factors (organic matter, electrical conductivity, pH, and lime). Soil maps were generated using interpolation techniques to visualize soil variation across the area. The sampling strategy was designed to ensure comprehensive data collection, allowing for robust model training and validation. MaxEnt, which is a presence-only model, outperformed both the GARP and logistic regression models in predicting suitable habitats for *Astragalus* sp. Results revealed that soil salinity, elevation, and soil acidity significantly influenced species distribution. The findings also suggest that elevation and salinity have the most substantial effects on habitat suitability, while soil texture (clay, silt, and sand) plays a secondary role. These results are valuable for rangeland management, offering insights into areas where *Astragalus* sp. could thrive or where interventions might be necessary to improve habitat conditions. In terms of management, this study highlights the importance of considering both ecological and environmental factors when planning conservation and restoration activities for rangelands. The ability to predict species distribution can help optimize resource allocation for habitat restoration and enhance biodiversity conservation efforts.

## 1. Introduction

Rangelands are among the most important and vast terrestrial ecosystems. These natural ecosystems have a vital role in the energy cycle, water storage, oxygen production, carbon sequestration, erosion control, preservation of biodiversity, wildlife, economics, society [1], and the protection and conservation of water and soil resources [2]. Changes in environmental characteristics are among the most important factors that control the spatial changes of vegetation; thus, knowing the diversity and distribution of changes in environmental factors and plants among different plant communities is necessary to achieve better management and to investigate the change process [3, 4]. The basic questions raised in the distribution of species, especially different species, are factors affecting the distribution and knowledge of the statistical and mathematical distribution model and the ecological needs of different species in the region. The current project mainly aims to investigate the geographical distribution model of the region's main species of diversity and provide comprehensive statistical models and relationships to identify the important habitats of diversity in the region. Recently, different modeling methods have been considered to find important influencing factors in the establishment and distribution of the species, which can determine its preferential tendency toward environmental factors [5]. Pearson and Dawson [6] evaluated climate models' effectiveness in predicting climate change's effects on species distribution. They stated that these models can be highly useful for finding a general view, but management factors, especially land use, should also be taken into consideration for a detailed examination of the habitats. Araujo et al. [7] studied and validated climate and species relationship models under climate change conditions. In a study of different climate models, Hijmans and Graham [8] concluded that the BioClim and maximum entropy (MaxEnt) statistical methods predict changes better than the DOMAIN and GAM methods. Therefore, using appropriate variables and factors influencing the presence of species is essential for sustainable rangeland management [9]. As mentioned, many ecological science researchers desire to identify the role of climatic factors in the distribution of species [10–12]. Sahragard, Ajorlo, and Karami [13] also investigated the artificial neural network, MaxEnt, and generalized linear model (GLM) to identify the habitat suitability of tree almond species in Fars Province. Their results indicated the appropriate performance of all three models on independent bioclimatic variables. Tanaka et al. [14] used two statistical models (i.e., GAM and multivariate adaptive regression splines (MARS)) to predict the potential habitat of species and found that species distribution models (SDMs) are useful tools for evaluating impacts on a large geographic scale and over long periods. Tóth et al. [15] studied the distribution of *Melitaea ornata* under these conditions, the last glaciation, and up to 2080, using the MaxEnt SDM. Their prediction results showed that temperate regions are less suitable for this species at present. Rana et al. [16] evaluated the geographical distribution of *Fritillaria cirrhosa* in Nepal using the MaxEnt model. The results revealed that the greatest habitat suitability between different climate scenarios of 2050 will occur in representative concentration pathway 4.5. They also predicted the movement of the species to suitable climatic regions in the northwest. Kakehmami et al. [17] used MARS and GLM for modeling the distribution of Juniper species (*Juniperus excelsa M.Bieb*) in southern Ardabil and northern Zanjan provinces and found that due to the different algorithms used, it is more reliable to employ multiple prediction methods rather than a single one. Overall, the literature reflects a trend toward integrating ecological knowledge with statistical methods to enhance understanding and management of plant species distributions in changing environments [18].

*Astragalus* sp., a genus of plants in the legume family, is of great ecological and economic importance. Known for its role in soil stabilization, nitrogen fixation, and as a forage plant for livestock, *Astragalus* sp. also contributes to biodiversity in rangeland ecosystems. Several species within this genus are also used for medicinal purposes, adding to their significance in human health. Understanding the distribution and habitat preferences of *Astragalus* sp. is crucial for effective rangeland management, conservation, and restoration efforts. Modeling the habitat of *Astragalus* sp. allows for the identification of suitable areas for its growth, which can be critical for sustainable land-use planning. Habitat modeling helps predict the species' potential distribution in response to environmental variables, such as soil properties, topography, and climatic factors. Given the increasing challenges posed by climate change and land-use alterations, habitat models can provide valuable insights into future habitat suitability under changing conditions. Ecological models for species distribution can be broadly classified into linear and nonlinear models. Linear models, such as logistic regression, assume a direct, proportional relationship between the dependent variable (species presence/absence) and the independent environmental variables. These models are relatively simple and interpretable but may not capture complex, nonlinear interactions in the data. On the other hand, nonlinear models like MaxEnt and Genetic Algorithm for Rule-set Production (GARP) can model complex, nonlinear relationships between species distribution and environmental variables, offering greater flexibility and predictive power, especially when only presence data are available. In this study, we employed both types of models to predict the distribution of *Astragalus* sp. in the Savar-Abad basin rangelands. The MaxEnt model is a presence-only method, relying solely on recorded presence data to predict species occurrence across a larger landscape. In contrast, presence–absence models like logistic regression and GARP incorporate both presence and absence data, which can provide a more comprehensive understanding of species distribution. This distinction is important as each method has its strengths depending on the type of data available and the ecological questions at hand. The research background in the field of species distribution modeling has evolved significantly over the years. Early studies focused on climate-based models like BioClim, while more recent advancements have included machine learning approaches, such as MaxEnt, which offer more refined predictions based on a variety of ecological factors. Previous research has shown the effectiveness of MaxEnt in predicting species distribution, particularly for plant species in arid and semiarid environments similar to those found in the Savar-Abad basin [13]. In addition, studies comparing logistic regression and GARP models have indicated that these models can provide useful complementary insights into habitat suitability.

The necessity of this study lies in the growing need for precise and reliable tools for managing rangeland ecosystems in the face of climate change and land degradation. As such, the application of SDMs has become indispensable for identifying areas with high conservation value and for planning restoration interventions. This study's innovative approach involves comparing multiple modeling methods—MaxEnt, GARP, and logistic regression—to provide a comprehensive understanding of habitat suitability for *Astragalus* sp. and to evaluate the performance of each model in predicting the species' distribution. By incorporating various environmental factors, such as soil properties, and physiography, this research contributes valuable insights into rangeland management, helping guide restoration and conservation efforts.

## 2. Materials and Methods

### 2.1. Study Area

**Figure 1:**
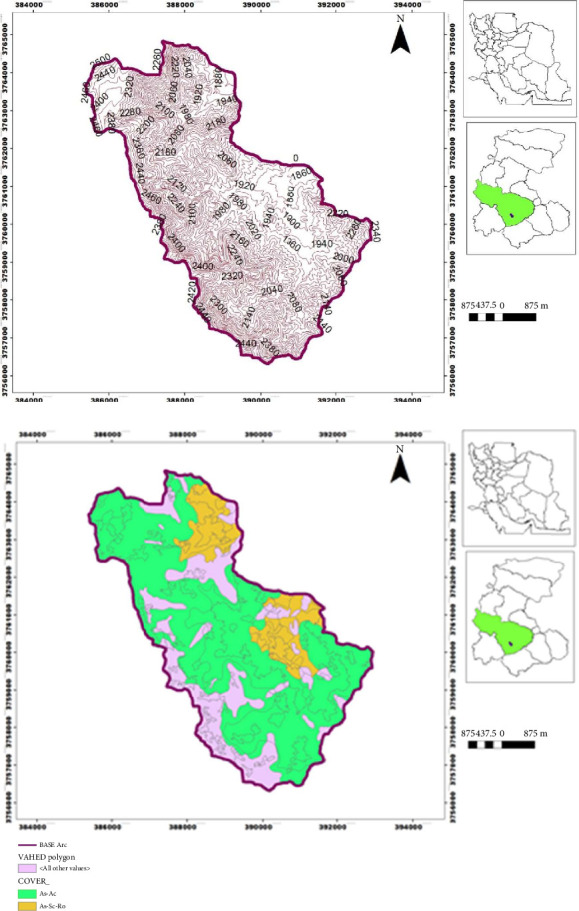
Location of the Savar-Abad basin in Markazi Province, Iran (a) and vegetation type map (b).

The basin of Savar-Abad, with an area equal to 3075 ha in terms of national divisions in the Markazi Province, is located between longitudes 49° 45′ 35″ to 49° 50′ 25″ longitude and 33° 56′ 35″ to 34° 01′ 23″ latitude (Figure 1). The highest elevation point is in the northwest of the region, with a height of 1840 m above sea level and an average slope of 36%. The average rainfall of the basin is 353 mm, and it is geologically located in the Sanandaj-Sirjan zone, mainly including low metamorphic limestone and slate rocks. The soil of the region is classified as entisol and inceptisol. This area's dominant type of vegetation belongs to the *Astragalus* sp.–*Acantholimon* sp. (*As-Ac*). The cropland area is mainly limited to the basin outlet, and rangeland is the dominant land use in the study area.

### 2.2. Field Investigation

In the studied area, the layer of slope, aspect, elevation classes, and vegetation types of different species were determined by performing field surveys and using topographic maps. Some sampling sites, with 2–10 square meters dimensions, were chosen in the homogeneous vegetation units. Information associated with the presence and absence of *Astragalus* (as a dependent variable) and other environmental information (as independent variables) underwent measurements [13, 18, 20].

The selection of 2- to 10-m^2^ plots for sampling is justified based on the need to accurately capture the diversity and distribution of plant species within the study area. This size allows for a representative sample of the vegetation types present, ensuring that various species can be adequately recorded and analyzed. Smaller plots may not encompass the variability of species and their interactions with environmental factors, while larger plots could introduce complexity and make it difficult to assess individual species' presence and abundance. The chosen size strikes a balance between these considerations, facilitating effective data collection. The study area consists of heterogeneous vegetation types, and using plots within this range helps in identifying the specific ecological requirements of *Astragalus* sp. and other associated species. This is crucial for understanding their distribution patterns in relation to soil and topographic characteristics; additionally, the minimum surface method used to determine plot size ensures that the sampling is tailored to the type and distribution of plant species, further validating the appropriateness of the 2- to 10-m^2^ range for the ecological context of the research.

A map of homogeneous units was prepared using satellite images based on the slope and height maps obtained from the digital elevation model (DEM) map with an accuracy of 10 m. Sampling near these areas was avoided due to the marginal effects of industrial areas, agricultural lands, and residential areas. Three transects of 750 m, two transects along the most dominant gradients (height, direction, and slope), and one transect perpendicular to those two transects are established in each homogeneous unit. Along each transect, some plots were placed at a distance of about 50 m; thus, 47 plots were established in each homogeneous unit (2 units and 94 plots in total). The size of the sampling plots was calculated according to the type and distribution of plant species using the minimum surface method. The type and number of plant species and their coverage percentage were recorded in each plot. For soil sampling, soil profiles were dug at the beginning and end of each transect, and soil sampling was conducted in line with the existing standards. Soil variables, including clay, gravel, silt, sand, organic matter (OM), electrical conductivity (EC), lime, and acidity, were analyzed in the laboratory. Furthermore, direction, longitude, latitude, slope, and height from the sea level were recorded in each sampling unit. In this study, dependent variables are the data on the presence and absence of vegetation types, which are marked with a code of zero and one, and the independent variables include soil and topography characteristics. The best distribution model was determined using statistical analysis.

### 2.3. Vegetation Sampling

Vegetation sampling was conducted across the Savar-Abad basin, with the aim of capturing the spatial distribution of *Astragalus* sp. and other plant species. The sampling design involved placing plots of 2–10 square meters in size within different vegetation types across the study area.

### 2.4. Soil Sampling

Soil sampling was carried out to measure key soil properties, which are critical in determining the habitat suitability of *Astragalus* sp. The sampling depth varied across the area depending on the soil profile but typically ranged from 0 to 30 cm. This depth was chosen as it represents the active root zone for many plant species, ensuring that soil factors affecting plant growth are accurately captured. Soil samples were taken from both the beginning and end of each transect to account for potential variation in soil properties along the gradient. Soil factors analyzed included physical properties such as sand, silt, clay, and gravel, as well as chemical properties such as OM, EC, pH, and total neutralizing value (TNV). These properties were selected due to their known influence on plant growth and their ability to vary significantly across different rangeland types. Soil maps for the study area were generated using geostatistical interpolation methods such as Kriging, to visualize the spatial variation of these soil factors. Kriging was chosen due to its robustness in estimating unknown values based on known data points and its ability to account for spatial autocorrelation.

### 2.5. Physiographic Factors

Physiographic data were collected to quantify the topographic features of the study area. These factors included slope, aspect, and elevation, all of which are crucial in determining plant distribution in mountainous and semiarid environments like the Savar-Abad basin. Slope and aspect data were derived from topographic maps and a DEM, with a 10-m resolution for high accuracy. Elevation data were collected at each sampling site using GPS coordinates. These physiographic factors, in combination with soil properties, provide a comprehensive view of the environmental factors influencing *Astragalus* sp. distribution.

### 2.6. Modeling Methods

Three SDMs were applied to predict the potential habitat of *Astragalus* sp.:

• Logistic Regression (Presence–Absence Model): Logistic regression is a widely used model for species distribution where both presence and absence data are available. It estimates the probability of the occurrence of a species based on the relationship between the species' presence and the environmental variables. The model was run using the maximum likelihood method, where coefficients for each independent variable were calculated to determine the probability of occurrence at each sampling site.
• MaxEnt (Presence-Only Model): The MaxEnt model, based on the MaxEnt principle, was used as a presence-only method to predict species distribution. This model only requires presence data and estimates the potential distribution based on environmental variables. The model was executed using the MaxEnt software (version 3.3.3e) with environmental layers (soil and physiographic factors) and species presence points in ASCII and CSV formats, respectively. The data were split into training (70%) and testing (30%) sets to evaluate the model's performance.
• GARP (Presence-Absence Model): The GARP model was used as an additional presence–absence model. This model generates distribution predictions by evolving rules based on environmental data and species presence/absence records. It was executed with 50% of the data used for training and the remaining 50% for evaluation, with the model being run 250 times to ensure consistency.

### 2.7. Model Validation

Each of the models was evaluated using a range of performance metrics. The ROC (Receiver Operating Characteristic) curve and AUC (area under the curve) were used to assess the accuracy of the predictions. An AUC value greater than 0.7 indicates good predictive power, while values above 0.9 suggest high accuracy. Additionally, the Kappa index was used to measure agreement between the predicted and observed species distributions. The Kappa index compares the classification of presence and absence in the model against observed data, with values closer to 1 indicating better performance.

### 2.8. Sensitivity Analysis

A sensitivity analysis was performed to identify the most influential environmental variables affecting *Astragalus* sp. distribution. This was done by running the models with each variable individually excluded and observing the effect on the model's accuracy. The Jackknife method in MaxEnt was used to determine the contribution of each environmental variable to the model's predictions.

## 3. Results and Discussion

The statistical characteristics of variables, including minimum, maximum, variance, and the like, are summarized in Table 1.

### 3.1. Results of Logistic Regression

The chi-square value for the Hosmer–Lemeshow goodness-of-fit test was 1.528, and the test was not significant (*p*=0.216), indicating the good validity of the model (Table 2).

The classification table for the selected final model is equal to 83% correct prediction of nonattendance points and 40.4% correct prediction for presence points, which is 61.7 in total (Table 3).

The classification Table 3 reports a 2 × 2 table that displays the numbers of correctly classified values at the user-specified cutoff. This table has four entries that report the number of observed 0 s (and 1 s) that were correctly (and incorrectly) predicted. Additionally, the classification table will provide information on the total number of observed 1 and 0 s, the total number of predicted 1 and 0 s, the percent of correctly classified 1 and 0 s, and the percent of total correctly classified observations.

The final model obtained in the backward method is model number 8, which demonstrates the significance of soil pH, which positively affects the dependent variable. The results of the logistic regression model with a predictor variable are presented in Table 4.

**Figure 2:**
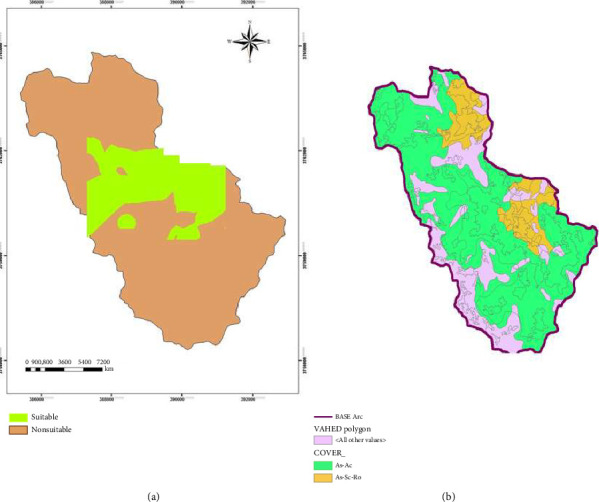
Prediction map of suitable areas of *Astragalus* sp. in Savar-Abad (rangelands Markazi Province, Iran) based on the logistic regression model (a) and vegetation type map (b).

According to the logistic regression model, increasing soil acidity could increase habitat suitability for different species. A suitable area map for *Astragalus* sp. was produced based on the logistic regression prediction algorithm. See Figure 2 for prediction map by selected model.

### 3.2. Results of the MaxEnt Model

After modeling, given that the output of the model is a continuous probability map, it is necessary to determine the presence or absence of the desired species. The presence and nonpresence of the species, its transformation, and the degree of its compatibility with the ground reality map were investigated by calculating the Kappa coefficient.

**Figure 3:**
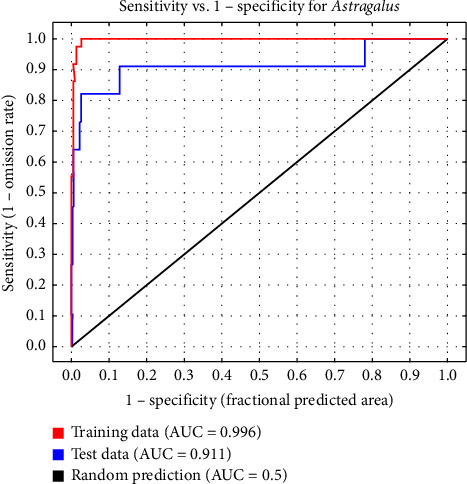
ROC curve of the MaxEnt model application in the study area. *Note:* AUC, area under the curve; MaxEnt, maximum entropy; ROC, receiver operating characteristic curve.

**Figure 4:**
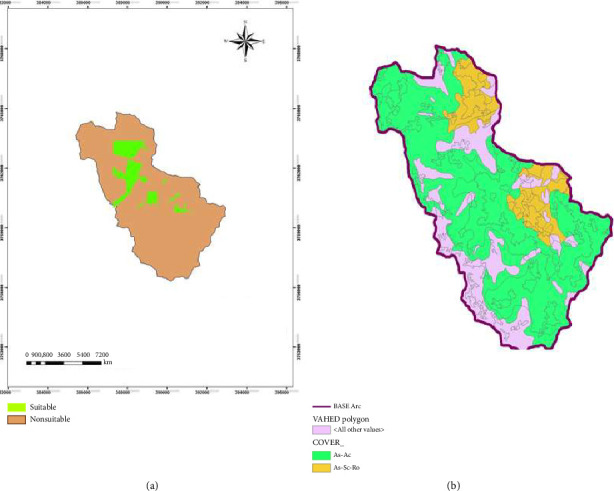
Prediction map of suitable areas of *Astragalus* sp. in the Savar-Abad rangelands (Markazi Province, Iran) based on the MaxEnt model (a) and vegetation type map (b). *Note:* MaxEnt, maximum entropy.

In Figure 3, the area under the prediction curve for the MaxEnt model is estimated in three cases. The model has good validity according to the area under the ROC curve in both cases calculated for the model creation data and the data test. Suitable and nonsuitable areas are depicted based on MaxEnt results in Figure 4.

### 3.3. Examination of the Importance of Variables in the MaxEnt Model

**Figure 5:**
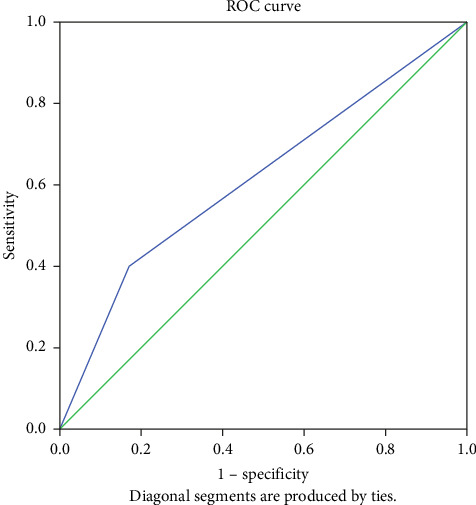
The area under the curve for the logistic regression model.

**Figure 6:**
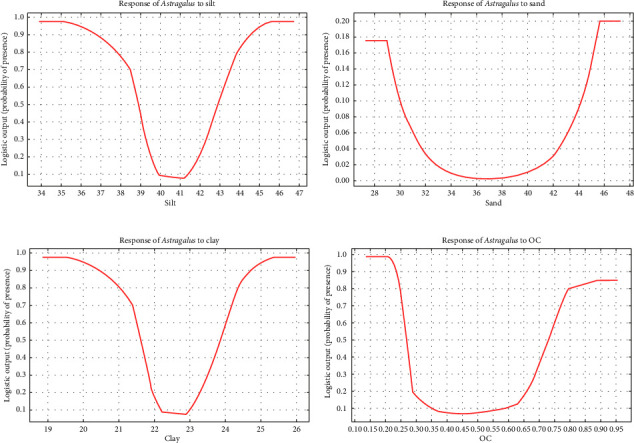
The response of *Astragalus* sp. to different environmental factors in the study area based on the MaxEnt model: (a) silt, (b) sand, (c) clay, and (d) organic content.

**Figure 7:**
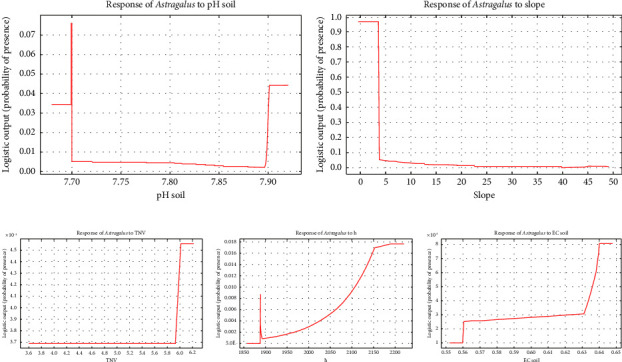
Response of *Astragalus* sp. to different environmental factors in the study area based on the MaxEnt model (a) pH, (b) slop, (c) soil TNV, (d) elevation, and (e) soil EC. *Note:* EC, electrical conductivity; MaxEnt, maximum entropy; TNV, total neutralizing value.

The sensitivity analysis curves of the corresponding model were used to control the importance of the variables in the MaxEnt model. Understanding the specific variables that influence habitat suitability is crucial for accurately modeling the distribution of *Astragalus* sp. The sensitivity analysis (Figure 5) indicates that removing salinity from the model leads to a substantial decrease in the area under the ROC curve (AUC), from 1.13 to 1.40. This suggests that salinity plays a critical role in determining suitable habitats for the species, and its exclusion can lead to less reliable predictions of habitat suitability. Altitude: While altitude is also an important variable, its removal results in a smaller decrease in AUC, from 1.24 to 1.20. This indicates that altitude influences habitat suitability but to a lesser extent than salinity. Understanding how altitude affects species distribution can help in identifying areas where *Astragalus* sp. may thrive or struggle. Soil Acidity: The logistic regression model indicates that increasing soil acidity can enhance habitat suitability for different species, including *Astragalus* sp. This highlights the importance of soil characteristics in determining the presence of the species. In summary, salinity, altitude, and soil acidity are critical variables influencing habitat suitability for *Astragalus* sp., and their careful consideration is essential for effective modeling and management strategies. Removing key variables such as salinity and altitude from habitat suitability models can significantly impact the accuracy and reliability of predictions. Salinity is identified as a crucial factor influencing the distribution of *Astragalus* sp. When salinity is excluded from the model, the area under the ROC curve (AUC) decreases from 1.13 to 1.40. This substantial drop indicates that the model's predictive power is significantly compromised, leading to less reliable assessments of suitable habitats for the species. The results suggest that salinity is essential for understanding the ecological needs of *Astragalus* sp. and its potential habitats. The removal of altitude results in a smaller decrease in AUC, from 1.24 to 1.20. This indicates that while altitude does affect habitat suitability, its influence is not as strong as that of salinity. The findings suggest that altitude still plays a role in determining suitable habitats, but its exclusion may not drastically alter the model's predictions. Understanding the relationship between altitude and species distribution is still important for habitat management. In summary, excluding these variables can lead to a misrepresentation of habitat suitability, potentially hindering effective conservation and management strategies for *Astragalus* sp. See Figures 6 and 7 for the response of *Astragalus* sp. to different environmental factors in the study area based on the MaxEnt model.

As shown, EC, or soil salinity (EC soil), increases the suitability of the habitat by increasing the value from 0.56. Moreover, the *Astragalus* sp. habitat's desirability increases by increasing the elevation value (h) from 1900 m. Similarly, the desirability of the *Astragalus* sp. habitat of different species increases by increasing the value of organic content from 0.3. Based on the data in Figures 6 and 7, the percentage of importance of acidity in predicting the habitat is different from other parameters, and the desirability of the *Astragalus* sp. habitat increases with an increase in acidity from 7.9. Sand in the MaxEnt analysis showed that desirability increases and then decreases with an increase in the percentage of soil sand from 38% to 44%. Considering the sensitivity curve of the soil texture parameter, it was determined that the percentage of silt has no effect on the habitat suitability of *Astragalus* sp. The area's topographic slope value as an environmental parameter demonstrated that habitat desirability decreases with an increase in the slope from 5%.

**Figure 8:**
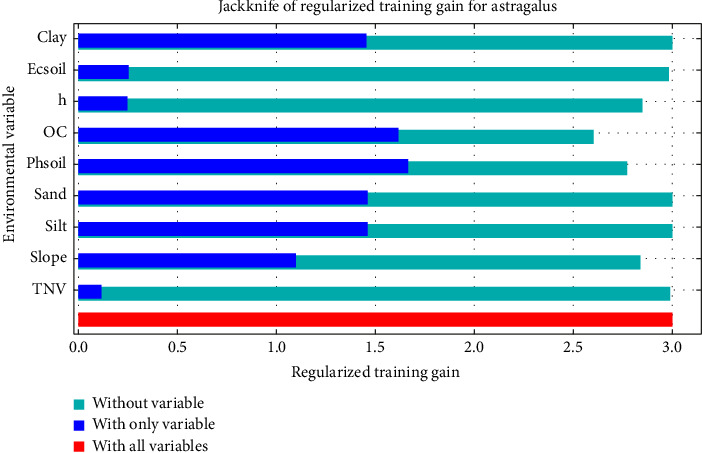
Percentage of the importance of different parameters based on the Jackknife index in predicting the suitability of the habitat of *Astragalus* sp.

The curve of neutralizing soil lime percentage in predicting the desirability of the *Astragalus* sp. habitat shows that the desirability of the habitat increases by increasing the value of TNV from 5.9 (Figures 6 and 7). According to the Jackknife test (Figure 8), the most important predictive variable of the soil acidity model is followed by the variable of soil OC. The OC variable provides the most unique information. Additionally, examining the contribution of variables indicates that OC and clay percentage are the most and the least important variables, respectively.

The area under the curve for the logistic regression model is lower than the area under the curve obtained for the MaxEnt model, so the validity of the MaxEnt model is higher (Table 5). The Jackknife test shows the achievement of addition in three different modes (i.e., without variables, with only one variable, and with all variables). This test demonstrates which of the variables has the most impact on the prediction of the model. The response curves of the variables are also drawn from the species distribution.

### 3.4. Modeling Species Distribution With the GARP Genetic Algorithm

**Figure 9:**
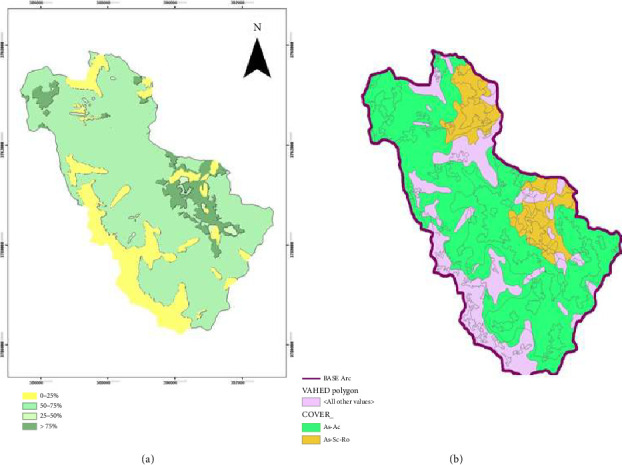
Map of the probability of the predicted species of *Astragalus* sp. with the GARP model in the Savar-Abad region (a) and vegetation type map (b). *Note:* GARP, genetic algorithm for rule-set production.

**Figure 10:**
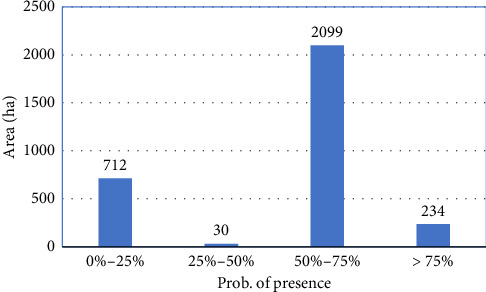
The area of the probability groups of *Astragalus* sp. species in the study area.

The results of the prediction map revealed the distribution ability of *Astragalus* sp. using the GARP model. The probability values of this map change from zero to one, and numbers close to one indicate the high probability of occurrence of the species. Based on the map generated from the total studied area, 7.6% of the area had a probability of occurrence of more than 75%, which is shown in the dark green color in Figure 9, and the light green areas represent places with a minimum probability of occurrence of species distribution less than 50%.  Figure 10 depicts the area of the different occurrence classes of the species in the region. The results of the evaluation of the model based on independent data and an error matrix showed that the predicted model with a Kappa coefficient value of 0.42 matches reality well. The area under the ROC plot curve equal to 0.78 indicates the good detection power of the model and confirms that the model had a good performance in predicting the species distribution. The results of sensitivity measurement and determination of variables affecting species distribution revealed that salinity and altitude are the most important environmental factors affecting species distribution (Figure 9), so that when the salinity factor is removed from the modeling process, the area under the ROC curve decreases from 0.75 to 0.48. However, if the height factor is removed, the area under the ROC curve decreases by only 0.2 and reaches 0.69, showing the greater effect of salinity on species distribution.

The comparison of different statistical models used to predict the spatial distribution of *Astragalus* sp. reveals significant differences in their predictive power. The MaxEnt statistical model demonstrated superior performance compared to both the genetic algorithm (GARP) and logistic regression models. The area under the curve (AUC) for the MaxEnt model was higher, indicating a better fit and predictive accuracy for the species distribution. The AUC for the logistic regression model was notably lower than that of the MaxEnt model, suggesting that logistic regression may not capture the complexities of the ecological data as effectively. This difference in AUC values highlights the statistical significance of the MaxEnt model's ability to predict habitat suitability more accurately than logistic regression. While the GARP model was chosen for its ease of use and reasonable performance, it still did not outperform the MaxEnt model. The statistical evaluation indicates that GARP may be less effective in certain ecological contexts, emphasizing the importance of selecting the right model based on the specific characteristics of the data. In conclusion, the statistical significance of differences between these models underscores the necessity of using robust modeling techniques like MaxEnt for accurate predictions in ecological studies.

### 3.5. Interpolation Methods for Soil Maps

Soil maps for the Savar-Abad basin were created using geostatistical interpolation methods, specifically Kriging, to estimate the spatial distribution of key soil properties. The interpolation methods used in this study help in predicting soil values at unsampled locations by accounting for spatial autocorrelation. The Kriging method was chosen due to its ability to provide unbiased predictions with quantifiable uncertainty, making it an effective tool for soil mapping. A variogram analysis was performed to examine spatial autocorrelation in the soil properties. The variograms showed a strong spatial dependence for most soil factors, particularly for soil OM and EC, indicating that these properties exhibit clear patterns across the study area. The interpolation methods, along with the calculated variograms, are summarized in Table 6.

### 3.6. Model Performance Evaluation

The performance of the three models was assessed using ROC curves and area under the curve (AUC). These metrics provide an indication of how well each model predicted the presence of *Astragalus* sp. in the study area. MaxEnt (Presence-Only Model): The AUC for the MaxEnt model was 0.93, indicating a very high predictive accuracy. The ROC curve for this model demonstrated strong performance, particularly in identifying suitable habitats for *Astragalus* sp., with well-defined distinctions between suitable and unsuitable areas. The AUC for the logistic regression model was 0.74, which indicates a moderate predictive ability. The ROC curve showed that while the model successfully predicted some areas of suitability, its accuracy was lower than that of MaxEnt, particularly for areas with extreme environmental conditions (e.g., high elevation and high salinity). The AUC for the GARP model was 0.80, indicating a good fit, but not as robust as the MaxEnt model. The GARP model was able to predict the distribution of *Astragalus* sp. well in areas with moderate environmental conditions, but it had difficulty in accurately predicting habitats in extreme conditions (e.g., very dry areas with low soil OM). The Kappa index for model comparison further validated these results. The Kappa values were 0.74 for MaxEnt, 0.48 for logistic regression, and 0.55 for GARP, indicating good agreement between predicted and observed species presence for MaxEnt and moderate agreement for the other two models.

### 3.7. Sensitivity Analysis

The sensitivity analysis (Table 7) showed that soil salinity (EC), elevation, and soil pH were the most influential variables affecting *Astragalus* sp. distribution. Removing salinity from the MaxEnt model caused a significant decrease in the AUC (from 0.93 to 0.77), highlighting its critical role in habitat suitability. Similarly, elevation and soil pH contributed substantially to the predictive accuracy, with smaller decreases in AUC when removed (from 0.93 to 0.88 and 0.90, respectively).

### 3.8. Distribution Maps

The predicted distribution maps for *Astragalus* sp. generated by the three models are shown in the above figures. These maps illustrate areas of high and low habitat suitability based on the different environmental variables.

• MaxEnt Map: Areas with high suitability (shown in dark green) correspond to regions with lower elevation, moderate salinity, and higher soil OM.
• Logistic Regression Map: The map shows more fragmented areas of suitability, with moderate accuracy in identifying the core habitat areas.
• GARP Map: The model's predictions show overlapping regions with those predicted by MaxEnt but with lower overall accuracy in extreme environmental conditions. The maps, along with the corresponding suitability scores, are crucial for understanding how environmental factors influence the distribution of *Astragalus* sp. and for guiding conservation and land-use decisions.

## 4. Conclusion

By comparing the elevation map and the final species presence map, it was found that the presence of the species has an inverse relationship with the elevation, and the presence of the species decreases with the increase in height. In general, each type of plant has a relationship with some factors of soil, elevation, and lowland, according to the characteristics of the growing area, ecological needs, and range of tolerance. Identifying how these environmental factors relate to species distribution can help rangeland managers manage rangelands, protect water and soil, and improve and revive rangelands [2, 20–23]. The GARP model is one of the correlation profile models that was chosen in this research due to its ease of use and proper performance. Correlation models do not have cause-and-effect relationships between variables. They can only predict the probability of occurrence of the studied species. Users with complementary ecological information can make judgments about the environmental factors affecting the species or ecological assumptions such as the effect of climate change or change of use on studying the distribution of plant species. By conducting similar research, factors affecting the establishment of plant species can be identified and used in pasture improvement and restoration programs. In addition, the presence of plant species in different areas can be predicted by using plant species habitat modeling. On the other hand, knowing the environmental factors in each region makes it possible to predict the probability of success or failure of establishing a plant species [20, 24]. Determining a set of variables that affect the distribution of plant species is important from the point of view through which they show their role in the species' biology and their adaptation to the limiting factors of the species' growth.

How individual species in plant communities respond to resources is important to know how environmental factors affect their distribution, abundance, and togetherness and to understand how different species function in a system [10, 25–27]. Zare Chahoki et al. [20] described MaxEnt as an accurate method for predicting the potential habitat of plant species. This method does not have many complications related to methods that use attendance and nonattendance data because it only uses attendance data for modeling. This study demonstrates the effectiveness of SDMs in predicting the habitat of *Astragalus* sp. in the Savar-Abad basin, using three distinct modeling approaches: MaxEnt, logistic regression, and GARP. Among these, the MaxEnt model—a presence-only model—provided the most accurate predictions, outperforming both logistic regression and GARP in terms of AUC and Kappa index. The sensitivity analysis identified soil salinity (EC), elevation, and soil pH as the primary environmental factors influencing *Astragalus* sp. distribution, reinforcing the importance of these variables in shaping habitat suitability in rangeland ecosystems. The differences in model performance highlight the strengths and limitations of each modeling approach. While MaxEnt excels in presence-only data scenarios, logistic regression remains valuable when both presence and absence data are available, and GARP offers flexibility in capturing complex ecological relationships. The application of these models provides valuable insights into the spatial distribution of *Astragalus* sp., which can inform conservation and restoration strategies in the study area. The findings underscore the significance of understanding the ecological factors influencing plant species distribution, particularly in the context of rangeland management. By identifying key drivers of habitat suitability, land managers can make more informed decisions about where to allocate resources for conservation and restoration. The ability to predict future changes in habitat suitability under climate change scenarios further enhances the utility of SDMs for proactive management. While the study has limitations, such as the relatively small sample size, the results offer promising directions for future research. Larger sample sizes and the inclusion of temporal data could improve the reliability of predictions and help refine models for better accuracy. Future studies could also explore the integration of remote sensing data to enhance spatial resolution and better capture environmental heterogeneity. In conclusion, the integration of SDMs into rangeland management provides an innovative approach to understanding and managing plant communities in a changing environment. The insights gained from this research can contribute to more effective and sustainable rangeland conservation strategies, supporting the long-term preservation of biodiversity and ecosystem services.

This study has several limitations. First, the sample size, though adequate for initial modeling, was relatively small, potentially limiting the generalizability of the results. Second, the geographic focus on the Savar-Abad basin may not fully represent the ecological conditions of other regions where *Astragalus* sp. occurs. Third, potential biases in data collection, such as uneven sampling across vegetation types or environmental gradients, could have influenced the results. Additionally, the models assume that the selected environmental variables are the primary drivers of species distribution, potentially overlooking unmeasured factors like biotic interactions or historical land-use changes. The models used (MaxEnt, GARP, and logistic regression) also have inherent limitations. For instance, MaxEnt may overestimate habitat suitability in under-sampled areas, while logistic regression assumes linear relationships that may not reflect complex ecological dynamics. These limitations highlight the need for caution in interpreting results and suggest future research should expand sample sizes, include broader geographic areas, and integrate additional data sources like remote sensing or long-term monitoring to improve model accuracy and applicability.

## Figures and Tables

**Table 1:** Description of variables included in the statistical models.

Variable	Number	Range	Minimum	Maximum	Average	Standard deviation	Variance
Presence	94	1	0	1	0.50	0.50	0.25
Soil erosion index	94	1	4.5	5.5	4.61	0.31	0.10
Soil hydrological group	94	1	1	2	1.11	0.31	0.10
Slope	94	41	4	45	27.85	16.02	256.58
Elevation	94	300	1890	2190	1992.16	89.62	8032.40
Aspect	94	6	1	7	3.18	1.80	3.25
Geology	94	1	1	2	1.11	0.31	0.10
EC	94	0.08	0.56	0.64	0.63	0.02	0.00
pH	94	0.2	7.7	7.9	7.81	0.07	0.00
Clay	94	6	19	25	22.21	1.67	2.79
Silt	94	11	35	46	40.81	2.97	8.85
Sand	94	17	29	46	36.68	4.67	21.77
Organic matter	94	0.75	0.2	0.95	0.43	0.22	0.05
TNV	94	2	4	6	5.59	0.54	0.29

Abbreviations: EC, electrical conductivity; TNV, total neutralizing value.

**Table 2:** Hosmer–Lemeshow test to improve the fit of the logistic regression model.

Step	Chi-square	Df.	Sig.
1	5.400	8	0.714
2	6.789	8	0.560
3	11.262	8	0.187
4	8.715	8	0.367
5	6.735	8	0.565
6	10.168	8	0.253
7	7.008	8	0.536
8	1.528	1	0.216

*Note:* Sig., significance level.

Abbreviation: df., degree of freedom.

**Table 3:** Prediction classification of the logistic model in the final step (8′im iteration).

Cases	Predicted (absence or 0)	Predicted (presence or 1)	Percentage correct
Observed (absence or 0)	28	19	40.4
Observed (presence or 1)	39	8	83.0
Overall percentage			61.7

**Table 4:** A summary of the fitted logistic regression model.

Variable	Coefficient	Wald	SE	Df.	Sig.
Constants	−56.3	4.6	26.14	1	0.03
pH soil	7.2	4.63	3.34	1	0.03

Note: Sig., significance level.

Abbreviations: df., degree of freedom; SE, standard error.

**Table 5:** Statistical analysis of the level under the forecast curve of the logistic regression model.

Area	Standard error^a^	Asymptotic significance^b^	Asymptotic 95% confidence interval	
			Lower bound	Upper bound
0.621	0.058	0.043	0.507	0.735

*Note:* The test result variable(s): Predicted probability has at least one tie between the positive and negative actual state groups. Statistics may be biased.

^a^Under the nonparametric assumption.

^b^Null hypothesis: True area = 0.5.

**Table 6:** The interpolation methods, along with the calculated variograms.

Soil factor	Interpolation method	Spherical model range (m)	Nugget (variance)	Partial sill (variance)	AUC value
Organic matter (OM)	Kriging	1000	0.12	0.85	0.92
Electrical conductivity (EC)	Kriging	900	0.09	0.78	0.91
Clay	Kriging	800	0.08	0.70	0.89
Sand	Kriging	850	0.07	0.65	0.87
Silt	Kriging	950	0.10	0.73	0.88

**Table 7:** Summary of the sensitivity analysis for key environmental variables.

Environmental variable	AUC (full model)	AUC (excluding variable)	% decrease in AUC (%)
Salinity (EC)	0.93	0.77	17.2
Elevation	0.93	0.88	5.4
Soil pH	0.93	0.90	3.2
Organic matter (OM)	0.93	0.91	2.1
Silt	0.93	0.91	2.1
Clay	0.93	0.92	1.1

## Data Availability

Datasets generated during the current study are available from the corresponding author upon reasonable request.
